# Comparative evaluation of the Sticky-Resting-Box-Trap, the standardised resting-bucket-trap and indoor aspiration for sampling malaria vectors

**DOI:** 10.1186/s13071-015-1066-0

**Published:** 2015-09-17

**Authors:** Katharina S. Kreppel, P. C. D. Johnson, N. J. Govella, M. Pombi, D. Maliti, H. M. Ferguson

**Affiliations:** Institute of Biodiversity, Animal Health and Comparative Medicine, University of Glasgow, Glasgow, UK; Environmental Health and Ecological Sciences group, Ifakara Health Institute, Dar-es-Salaam, Tanzania; Dipartimento di Sanità Pubblica e Malattie Infettive, Università di Roma “Sapienza”, Rome, Italy

**Keywords:** *Anopheles arabiensis*, Malaria vector, Resting behaviour, Exophily, Sticky trap, Ecology, Resting traps

## Abstract

**Background:**

Understanding mosquito resting behaviour is important for the control of vector-borne diseases, but this remains a challenge because of the paucity of efficient sampling tools. We evaluated two novel sampling methods in the field: the Sticky Resting Box (SRB) and the Resting Bucket trap (RBu) to test their efficiency for sampling malaria vectors resting outdoors and inside houses in rural Tanzania. The performance of RBu and SRB was compared outdoors, while indoors SRB were compared with the Back Pack Aspiration method (BP). Trapping was conducted within 4 villages in the Kilombero Valley, Tanzania over 14 nights. On each night, the performance for collecting *Anopheles* vectors and *Culicinae* was compared in 4 households by SRB and RBu outdoors and by SRB or fixed-time Back Pack aspirator in 2 of the 4 focal households indoors.

**Findings:**

A total of 619 *Anopheles gambiae* s.l., 224 *Anopheles funestus* s.l. and 1737 *Culicinae* mosquitoes were captured. The mean abundance of *An. arabiensis* and *An. funestus* s.l. collected with SRB traps inside and outdoors was significantly lower than with BP or RBu. The SRB however, outperformed BP aspiration for collection of *Culicinae* indoors.

**Conclusions:**

Of the methods trialled indoors (BP and SRB), BP was the most effective, whilst outdoors RBu performed much better than SRB. However, as SRB can passively sample mosquitoes over a week they could provide an alternative to the RBu where daily monitoring is not possible.

## Findings

Currently there are few reliable and widely applicable methods for studying the resting behaviour of Anopheles malaria vectors in outdoor and indoor settings. Conventional methods of collecting mosquitoes resting indoors include Pyrethrum Spray Catches (PSC) and active mouth/electric aspirations [1]. Current tools for sampling mosquitoes outdoors include Muirhead-Thomson Pit-Shelters [2], resting boxes [3, 4] or clay pots [5]. No standardised method for use indoors and outdoors is available, making it difficult to infer unbiased estimates of mosquito resting habitat preference from these current methods. This study investigated the performance of two lightweight, portable traps for collecting resting mosquitoes in rural Tanzania: the recently developed Sticky Resting Box (SRB) [6], a variant of the mosquito Resting Box [4], and the Resting Bucket (RBu), a new trap presented here. Both traps are inexpensive and easy to produce from materials readily available in rural African settings. These features would make it easy to deploy these traps in high numbers as part of routine vector surveillance.

## Methods

Mosquitoes were collected within 4 villages in the Kilombero Valley in south-eastern Tanzania: Kidugalo (S08°30.7258′; E036°31.8476′), Lupiro (S08°23.2956′; E036°40.6122′), Minepa (S08°16.4974′; E036°40.7640′) and Sagamaganga (S08°03.8392′; E036°47.7709′) where malaria is endemic. The dominant malaria vectors are *An. arabiensis* and *An. funestus* s.l. Trapping of resting mosquitoes was conducted indoors and outdoors at 4 households in each village over 14 nights between August and September 2012, coincident with the end of the dry season. In each village, the owner of a randomly selected house based on a 2012 IHI DSS census list and owners of 3 neighbouring houses (100–200 m distance) were asked to partake.

**Fig. 1:**
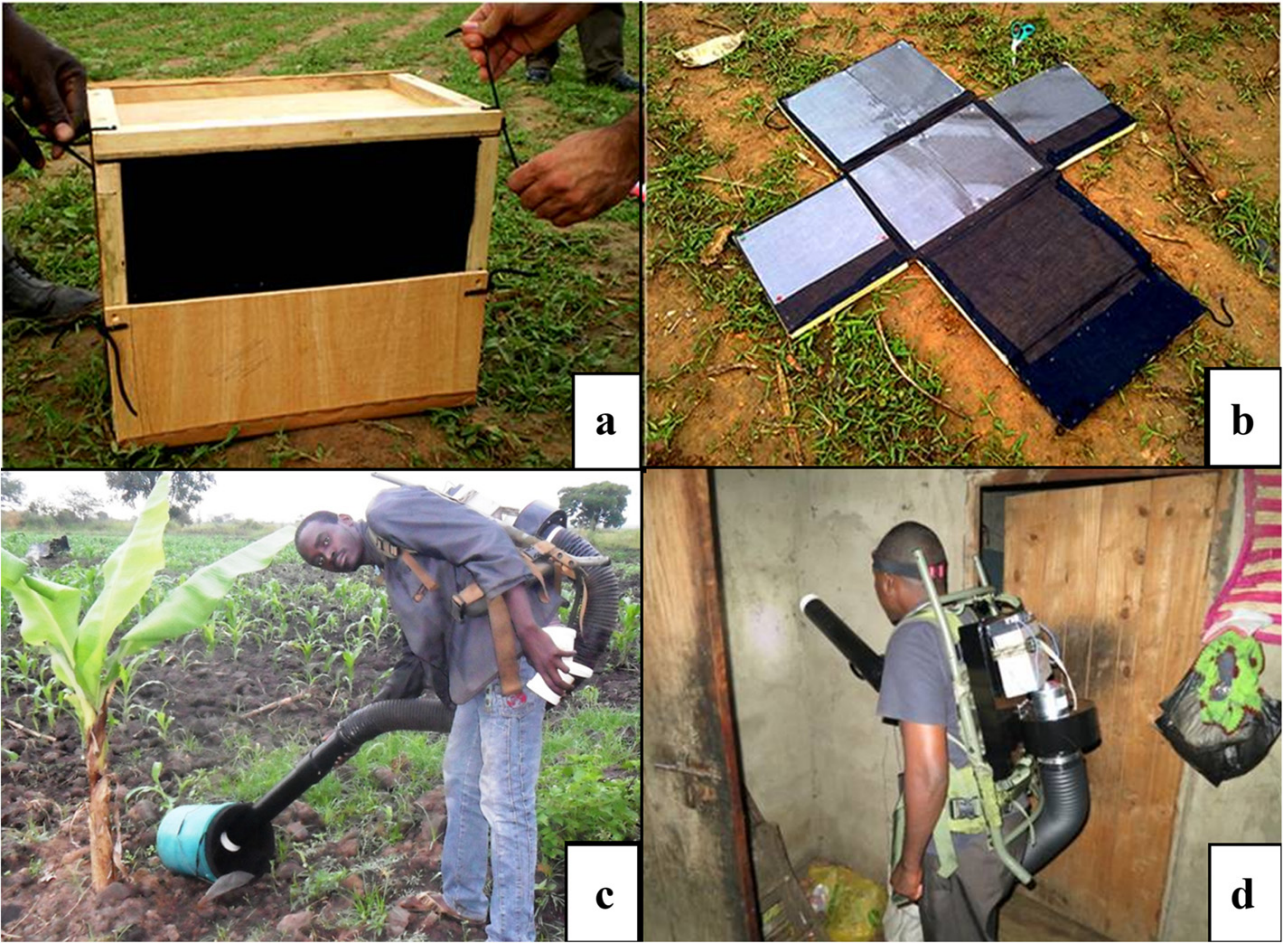
Trapping methods to collect resting mosquitoes **a**) Sticky Resting Box trap (30 cm × 60 cm × 30 cm) fully assembled, lined with black cloth **b**) Sticky Resting Box trap opened with six A4 acetate sheets covered in glue **c**) Resting Bucket trap made from a standard 20 l plastic bucket, lined with black cloth, aspirated by CDC backpack aspirator **d**) Standard battery-powered CDC Back pack aspirator (Model 1412, John Hock, Florida USA)

The SRB trap is - as described in Pombi et al. [6] - a rectangular wooden box, lined with black cotton cloth inside on top of which glue covered acetate sheets are attached to trap mosquitoes that enter to rest (Fig. 1a and b). During trapping it was placed on its side, and partially opened on one end to allow mosquitoes to enter. The other outdoor trap used was the RBu (Fig. 1c) made from a standard 20 l plastic bucket (290 mm diameter opening and 390 mm deep) also lined with black cotton fabric, and set by placing it on its side with the opening facing a house. Inside both traps a wet cloth was placed to increase humidity. Within the desired 5 m distance range from households, outdoor traps were positioned facing the house in relatively shady areas, ideally next to or under vegetation. Each night two SRBs and three RBus per household per village were set up outdoors. SRB traps were also used to trap mosquitoes resting indoors, with one trap being placed on the floor in the sleeping room or its entrance area. Indoor resting mosquitoes were also collected by sweeping a CDC Back Pack aspirator (BP, Model 1412, John Hock, Florida USA, Fig. 1d [7]) over the walls and ceiling of sleeping rooms for a 10 min period. All SRB and RBu traps were set at dusk. All SRB and RBu were checked and indoor BP aspiration conducted between 6.00-8.00 am the next morning. Mosquitoes resting inside RBus were collected using a CDC backpack aspirator and aspirating for 10–20 s at the open end of the bucket. Whilst only the mosquitoes resting inside RBu and SRB or on walls at the time of sampling would be collected, the sticky lining of the SRB meant that any mosquito resting in the trap during the preceding 12 h should have been caught.

All trap types were tested on all nights of the study. However, the two indoor collections per house per night (BP and SRB) could not be compared simultaneously in the same house because of their potential competition for mosquitoes within the same room. Consequently a modified Latin-square design was used in which only one indoor trapping method was used per house per day. On each night, one SRB was placed in a sleeping room of 2 houses, whilst BP aspiration was conducted in the remaining two. Over successive nights, trapping methods and collectors were rotated through households.

All mosquitoes caught were morphologically identified to genera [8] and a subset of those identified as belonging to the *An. gambiae* s.l. species group (*n* = 285, 46 % of the total) were identified to species level by PCR [9]. Before the study began, permission of community leaders in all four villages was sought and informed consent obtained from each head of household where trapping was conducted. Ethical approval and research clearance was obtained from the institutional review board of Ifakara Health Institute in Tanzania (IHI/IRB/No. A50) and the Medical Research Coordination Committee of the National Institute of Medical Research in Tanzania (NIMR/HQ/R.8c/Vol.ii/125).

Statistical analysis was conducted to compare the mean abundance of malaria vectors and *Culicinae* captured per night by each trapping method using the statistical software R [10]. Separate analyses were done for mosquitoes caught indoors (SRB vs BP) and outdoors (SRB vs RBu). Variation in the proportion of malaria vectors (combining *An. gambiae* s.l. and *An. funestus* s.l.) within the total mosquito catch was investigated. Generalised linear mixed effects models (GLMM, package lme4 in R) were used, fitting trap type as fixed effect, and night, date and house as random effects.

### Results and discussion

Forty six percent of the *An. gambiae* s.l. sample were molecularly analysed and all were identified as *An. arabiensis*. As this finding matches other observations indicating that >93 % of *An. gambiae* s.l. from the Kilombero Valley are *An. arabiensis* [11], it was assumed that all *An. gambiae* s.l. caught in this study represent *An. arabiensis*.

In total, 619 *An. gambiae* s.l. and 1737 *Culicinae* mosquitoes were captured (Table 1). All traps predicted similar geographical trends in mosquito abundance with numbers estimated as being highest in Lupiro, followed by Minepa and Kidugalo and lastly Sagamaganga.

**Table 1:** Total counts of mosquitoes collected by species per trap type regardless of number of traps

Mosquito type	Outdoors		Indoors	
	RBu	SRB	BP	SRB
*An. arabiensis*	514	17	83	5
*An. funestus* s.l.	152	3	69	0
*Culicinae*	443	490	734	70
Total	1109	510	886	75

Outdoors, the RBu collected significantly more malaria vector species per trap per night than the SRB (Table 2, >20–35 more *An. arabiensis* and *An. funestus* s.l*.,* respectively) while the SRB caught approximately 1.25 times more *Culicinae* than the RBu (Table 1). Controlling for random variation between villages, the RBu was estimated to be significantly more efficient at sampling *An. arabiensis* (*χ*^*2*^ = 15.01, *p* < 0.001) and *An. funestus* s.l*.* (*χ*^*2*^ = 13.3, *p* = 0.002) than the SRB, which in turn collected significantly more *Culicinae* than the RBu (*χ*^*2*^ = 4.99, *p* = 0.02; Table 2).

**Table 2:** Predicted mean abundance of mosquito groups per trap per night caught outdoors

Village	Predicted abundance of mosquitoes per trap per night outdoors					
	*An. arabiensis*		*An. funestus* s.l.		*Culicinae*	
	SRB	RBu	SRB	RBu	SRB	RBu
Kidugalo	*	0.03 (0.004-0.19)	*	0.39 (0.15-0.98)	0.0004 (40–0.34)	0.0003 (0–0.22)
Lupiro	0.12 (0.03-0.46)	3.71 (1.47-9.32)	0	1.52 (0.49-4.64)	12.11 (4.45-32.9)	7.21 (2.75-18.84)
Minepa	0.39 (0.2-0.75)	6.42 (4.42-9.31)	0.35 (0.16-0.74)	0.68 (0.2-2.29)	1.79 (0.56-5.68)	0.82 (0.27-2.45)
Sagagmaganga	*	0.16 (0.04-0.66)	*	0.004 (0–158.48)	0.000003 (0–0.05)	0.00007 (0–1)
Total	0.03 (0.002-0.32)	0.65 (0.06-6.69)	0.007 (0.002-0.02)	0.25 (0.11-0.54)	0.34 (0.03-3.05)	0.27 (0.03-2.42)

Numbers in brackets represent 95 % confidence interval. Asterisks indicate where no mosquitoes were caught

Indoors, the SRB was much less efficient in sampling *An. arabiensis* than the Back Pack Aspiration (BP), and failed to capture a single *An. funestus* s.l. (Table 1). Regardless of trap type, malaria vectors constituted a minority of indoor-resting mosquitoes (17 % or less, Table 1). The proportion of vectors estimated caught in BP collections was ~6 times higher than in SRB.

The BP caught approximately 20 times more *An. arabiensis* per collection than the SRB, 1.7 times more *An. funestus* s.l*.* and 24 times more *Culicinae* (Table 3), with significant differences for *An. arabiensis* (*χ*^*2*^ = 5.01, *p* = 0.02), *An. funestus* s.l. (*χ*^*2*^ = 6.73, *p* = 0.009) and *Culicinae* (*χ*^*2*^ = 6.37, *p* = 0.01).

**Table 3:** Predicted mean abundance of mosquito groups per trap per night caught indoors

Village	Predicted abundance of mosquitoes per trap per night indoors					
	*An. arabiensis*		*An. funestus* s.l.		*Culicinae*	
	SRB	BP	SRB	BP	SRB	BP
Kidugalo	*	0.16 (0.02-1.13)	*	1.84 (0.8-4.19)	*	1.85 (0.7-4.83)
Lupiro	0.83 (0.22-3.08)	9.33 (3.36-25.8)	*	4.3 (1.15-15.98)	10.5 (4.98-22.11)	52.9 (25.61-109.24)
Minepa	*	2.8 (0.88-8.89)	*	0.56 (0.08-3.89)	0.71 (0.18-2.74)	21.9 (7.59-63.11)
Sagamaganga	*	0.12 (0.01-0.85)	*	0.32 (0.02-4.09)	0.28 (0.02-3.79)	8.9 (3.54-22.35)
Total	0.04 (0–0.42)	0.82 (0.06-6.31)	*	1.77 (0.6-5.2)	0.31 (0.05-1.77)	7.52 (1.5-37.51)

Numbers in brackets represent 95 % confidence interval. Asterisks indicate where no mosquitoes were caught

Our finding that *An. arabiensis* was caught resting mainly outdoors is in line with its known exophilic behaviour [12–14], as was the observation that the more endophilic *An. funestus* s.l. [15, 16] was primarily caught indoors. However, the SRB only caught *An. funestus* s.l. outdoors, as also found in Burkina Faso [6]. Whilst SRB caught very few malaria vectors, it caught a much higher abundance of *Culicinae* than either RBu (outdoors) or BP (indoors), with the majority being gravid females. This is in agreement with findings from Burkina Faso, where SRB showed high performance in collecting *Culicinae* [6]. A potential explanation for the attraction of gravid *Culicinae* to the SRB could be the scent of the glue (polybutylene based adhesives are used in oviposition traps [17, 18]) which may mimic oviposition odour cues used by these species [19]. Findings such as by Lindh et al. [20] indicate that odour cues are very species specific. This is also supported by results from Thailand showing high *Culex spp.* collections with an oviposition trap for *Aedes* equipped with glue utilised in the SRB [21, 22]. The performance of the SRB for capturing *An. gambiae* s.l. indoors was relatively low here (~4 % relative to the BP) in comparison to what was found in a previous study in Burkina Faso (~16 %, [6]). Previous studies of sampling methods for mosquitoes have also shown high spatial variability in relative trap performance [23–26] which highlights the need to evaluate traps in different geographical settings. Potential reasons for the apparent differences in the performance of the SRB in Burkina Faso and Tanzania include differences in species composition within the *An. gambiae* s.l*.* complex and study design. While data was collected from two sites over 2 years in Burkina Faso, collections were made over 14 trap nights in Tanzania and fewer traps were used. The SRB was positioned facing the house in both studies, but in Burkina Faso the trap was just centimetres from the wall, while it was set at a distance of 5 m in Tanzania.

## Conclusion

We conclude that currently in the Kilombero Valley in Tanzania, the most efficient means among those tested of sampling resting malaria vectors is the RBu and BP for outdoor and indoor settings respectively, with the SRB performing considerably worse in both resting habitats. In addition to higher relative sampling efficiency, practical advantages of the RBu are its negligible impact on the environment relative to other methods and being the most convenient trap in terms of transport, set up and maintenance. However, despite lower relative sampling efficiency, the SRB retains unique features that could be advantageous. Specifically, the SRB alone can be used for passive sampling over several days without the need for regular checking, thus increasing its performance in terms of mosquitoes collected without an increase of human effort in the field [6]. Additionally, SRB could be recommended in studies where *Culicinae* are the primary target.

Within the Kilombero Valley where *An. arabiensis* is the predominant vector, RBu traps have shown to be an efficient, low cost sampling method for outdoor resting mosquitoes. We believe this method has strong promise to be further developed into a standardised sampling tool and encourage further investigation of its potential use in a wider range of African settings.

## Abbreviations

SRB: Sticky resting box
RBu: Resting Bucket
BP: Back pack aspiration
